# Automated Curb Recognition and Negotiation for Robotic Wheelchairs

**DOI:** 10.3390/s21237810

**Published:** 2021-11-24

**Authors:** Sivashankar Sivakanthan, Jeremy Castagno, Jorge L. Candiotti, Jie Zhou, Satish Andrea Sundaram, Ella M. Atkins, Rory A. Cooper

**Affiliations:** 1 School of Health and Rehabilitation Sciences, University of Pittsburgh, Pittsburgh, PA 15260, USA; sis65@pitt.edu (S.S.); jlc118@pitt.edu (J.L.C.); jiz156@pitt.edu (J.Z.); sas339@pitt.edu (S.A.S.); 2 US Department of Veterans Affairs, Pittsburgh, PA 15206, USA; 3 Robotics Institute, University of Michigan, Ann Arbor, MI 48109, USA; jdcasta@umich.edu (J.C.); ematkins@umich.edu (E.M.A.)

**Keywords:** obstacle negotiation, step-climbing wheelchair, rehabilitation engineering

## Abstract

Common electric powered wheelchairs cannot safely negotiate architectural barriers (i.e., curbs) which could injure the user and damage the wheelchair. Robotic wheelchairs have been developed to address this issue; however, proper alignment performed by the user is needed prior to negotiating curbs. Users with physical and/or sensory impairments may find it challenging to negotiate such barriers. Hence, a Curb Recognition and Negotiation (CRN) system was developed to increase user’s speed and safety when negotiating a curb. This article describes the CRN system which combines an existing curb negotiation application of a mobility enhancement robot (MEBot) and a plane extraction algorithm called Polylidar3D to recognize curb characteristics and automatically approach and negotiate curbs. The accuracy and reliability of the CRN system were evaluated to detect an engineered curb with known height and 15 starting positions in controlled conditions. The CRN system successfully recognized curbs at 14 out of 15 starting positions and correctly determined the height and distance for the MEBot to travel towards the curb. While the MEBot curb alignment was 1.5 ± 4.4°, the curb ascending was executed safely. The findings provide support for the implementation of a robotic wheelchair to increase speed and reduce human error when negotiating curbs and improve accessibility.

## 1. Introduction

Electric Powered Wheelchairs (EPWs) are essential assistive devices for people with mobility impairments. Approximately 500,000 people benefit from EPW in the U.S. This figure is expected to increase at a rate of 5% per year [1] due to new cases of spinal cord injuries and diseases. When performing activities of daily living, EPW users are often exposed to environmental barriers such as uneven terrain and inaccessible sidewalks especially when curb-cuts are not available, blocked, or in bad condition [2]. For example, Bennett et al. reported that only 2.6% of all street intersections met accessibility guidelines [3]. Driving over such environmental barriers may damage the EPW and injure users [4]. Most commercial EPWs claim to safely traverse small surface thresholds of up to 7.6 cm (3.0 inches) [5,6]. Despite these mobility benefits, many commercial EPWs are unable to drive over typical curbs of 0.15–0.21 m (6.0–8.0 inches) [7], leading to lower independence and participation of EPW users in the community.

Robotic Wheelchairs (RWs) are an emerging mobile assistive technology that can incorporate many degrees of freedom with innovative algorithms to overcome architectural barriers and limited accessibility [8]. The Stair Climbing Mobility System by the University of Castilla can ascend automatically average step heights by using legged wheel actuators. Prior to ascending steps, the user is required to align perpendicularly to the curb and ascend in reverse to shift the center of mass proportionally at each step [9]. Commercially available robotic wheelchairs such as the iBot can climb steps up to 0.13 m (5.0 inches) height in real-time [10]. Similarly, in the Stair Climbing system, the user is required to align with the curb before ascending in addition to pulling up from an external handrail to assist the iBot during the ascending process. The RT-Mover offers step-climbing capabilities for different step heights and angle approaches using legged wheel actuators and front-wheel photoelectric sensors to detect curb height. However, it is unclear about the minimum distance that the device must be away from the step to initiate the step-ascending process or detect the curb [11]. It is a common theme that RWs require a pre-planning phase before negotiating steps performed by the end-user. Aligning the RW to the step requires complete spatial awareness by the user; however, this may not be possible due to visual impairment, seat positioning, or limited trunk stability that could restrict visibility. RW misalignment could increase the risk of tips and falls while negotiating steps. The need for RWs to promote accessibility must be complemented by adequate sensing capabilities to detect environmental barriers, surrounding obstacles, and available surface area to ascend or descend steps or curbs for user’s safety and further implementation of RW capabilities.

Alternatively, research in smart wheelchairs has investigated add-on or built-in sensors on commercial EPWs for obstacle detection and avoidance [12]. Most of these devices use proximity sensors (e.g., ultrasound, sonar, LiDAR, or laser range finders) and pre-planning algorithms such as Simultaneous Localization and Mapping (SLAM) to characterize obstacles. Additionally, these sensors are optimal in ideal conditions, short distances, and focused on indoor environments. The authors identified three commercially available add-on devices: SmileSmartSystem (SmileSmartSystem, Berks, UK) and Braze Sentina (Braze Mobility, ON, CA, USA) for 2D obstacle recognition, and LUCI (LUCI Mobility, TN, USA) for 2D obstacles and recognizing surface irregularities (e.g., curb drop). However, the internal operations are not disclosed to establish their full capabilities and further implementation with RWs.

Sensors used for curb data acquisition can be separated into proprioceptive (internal), contact, and exteroceptive (external). Proprioceptive sensors are internal to the device for example the motor speed. Contact external sensors such as force could be resistive, capacitive, or optical sensors. Lastly, exteroceptive sensors could be cameras (monocular or stereo), LiDAR (2D/3D), ultrasonic, or a multimodal concept [13]. Romero et al. examined the history of road curb detections for vehicles to evaluate the different types of range sensors and methods used to identify road curbs [14]. These methods and technologies were evaluated based on the curb as a boundary between the road and sidewalk and treated as a non-negotiable obstacle. Ultrasonic sensors used to detect curbs have a range from a few centimeters up to 10m, but their main disadvantage is accuracy [15]. In the automotive industry, LiDAR is one of the most common sensors used for curb detection [16]. It emits pulses of laser light and is minimally affected by external lighting conditions, providing accurate distance measurements. However, they are large, expensive, and return incorrect results when affected by wet or black surfaces, and in some cases unable to provide enough context information for curb detection [16] or provide false positives [17,18]. Cameras work at high-frame rates providing dense information conditional to good illumination and weather. Additionally, stereo infrared cameras may be used to generate real-time depth data and may work better in low light conditions, especially coupled with an infrared emitter [19]. An alternative option is a multimodal sensor fusion approach where LiDAR and vison are combined. These methods improve the accuracy and detection of curbs but at a high cost of computational and equipment cost [20,21]. Computational costs include aligning the data streams of their temporal and spatial resolution, data format, and geometric alignment [22]. 

Curb detection can be separated into two categories, classification, and thresholding, where classification relies on machine learning, support vector machines, and neural networks [14], and thresholding relies upon the curb’s geometric features. El-Halawany et al. segment a point cloud as ground and nonground by examining the distance and slope of consecutive points and obtaining the elevation gradient and surface normals from the point cloud [23]. Yao et al. present a method where 3D LiDAR data are projected upon an *x*–*y* plane and the elevation of a point is compared to its neighbors to determine curb classification. All points classified as a curb are then fit to a single parabola using RANSAC, meaning multi-edge surfaces cannot be detected [24]. Alternatively, line fitting and identification methods such as the Hough transform can be used to detect lines in a raster image. A Canny edge detector [25] is first applied to high dimensional color or depth imagery to detect edges in the scene as a binary image. Then a Hough transform is applied to the binary image to extract the relevant lines which are clustered using a Hough accumulator [26]. Panev et al. relies upon detecting three distinct curb edges (ground, upper and rear corner edge) with a fisheye lens monocular camera used for autonomous cars. Their method utilizes the Hough transform for edge detection, and SVM for outlier rejection, and relies upon prior knowledge of the curbs geometry to match detected lines to a curb template. The curb detection was tested using a limited dataset and will not work near curb corners [27].

Although there have been numerous research studies for curb detection in the automotive industry, very few have been applied to the robotic wheelchair research to create an automated curb negotiation system. This study proposes a Curb Recognition and Negotiation (CRN) system to recognize curb dimensions and improve curb negotiation capabilities of RWs. The contributions of this study include:

- The first fully integrated open-source autonomous framework for a curb climbing robotic wheelchair was validated experimentally (Castagno and Sivakanthan 2021, https://github.com/sivashankar28/polylidar-realsense-wheelchair, (accessed on 20 October 2021)).
- A novel and robust curb-edge detection method that extracts a curb edge from the polygonal representation of the sidewalk surface. Standard line extraction methods are significantly more efficient by operating on a low-dimensional polygon instead of dense image space.
- An automated-driving three-step sequence that uses the curb characteristics to drive and negotiate using an existing curb climbing sequence.

The following sections describe the software development of the CRN system and its integration with an existing RW. An evaluation of the CRN system is discussed using an engineered curb at different angles of approach. The final section discusses the automated process, potential applications of the CRN system, and future work.

## 2. Materials and Methods

### 2.1. Previous MEBot Curb Recognition and Negotiation System

**Figure 1:**
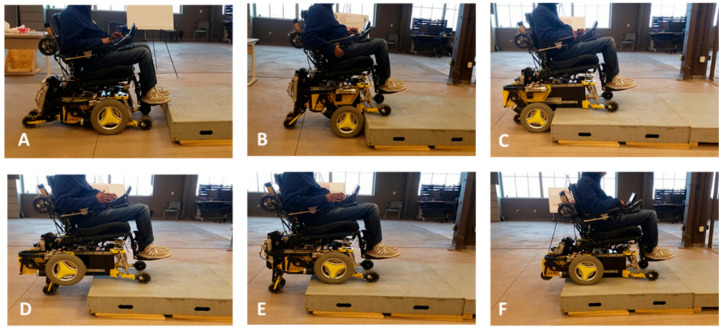
MEBot curb ascending process: (**A**) starting position; (**B**) the MEBot elevates and drives until front driving wheels touch the curb; (**C**) driving wheels move back, allowing the center of mass to shift onto the curb; (**D**) driving wheels are raised; (**E**) driving wheels move forward and onto the curb; and (**F**) driving carriage moves back whilst driving forwards and rear castors are raised until the MEBot is completely on the curb.

This section explains the curb negotiation capabilities of a mobility enhancement robot (MEBot), limitations, and the integration of the CRN system to improve the application. The MEBot was created as a testbed for advanced mobility applications to overcome environmental barriers [28]. The MEBot is a robotic wheelchair with six height-adjustable wheels and a modular drive wheel configuration. These features provide indoor/outdoor maneuvering and curb negotiation to improve accessibility. A previous study explained the sequential steps to ascend and descend curbs in an automated process (Figure 1) [29]. Similar to RWs previously discussed, the MEBot must meet two assumptions prior to curb negotiation: the wheelchair must be aligned perpendicular to the curb for full contact of the wheels with the curb, and the curb height and approach angle must be known. These procedures were performed by the user as described in Figure 1A. Usability evaluations with EPW users highlighted the challenges of curb alignment for safe negotiation [28].

### 2.2. CRN Hardware and Software Development

**Figure 2:**
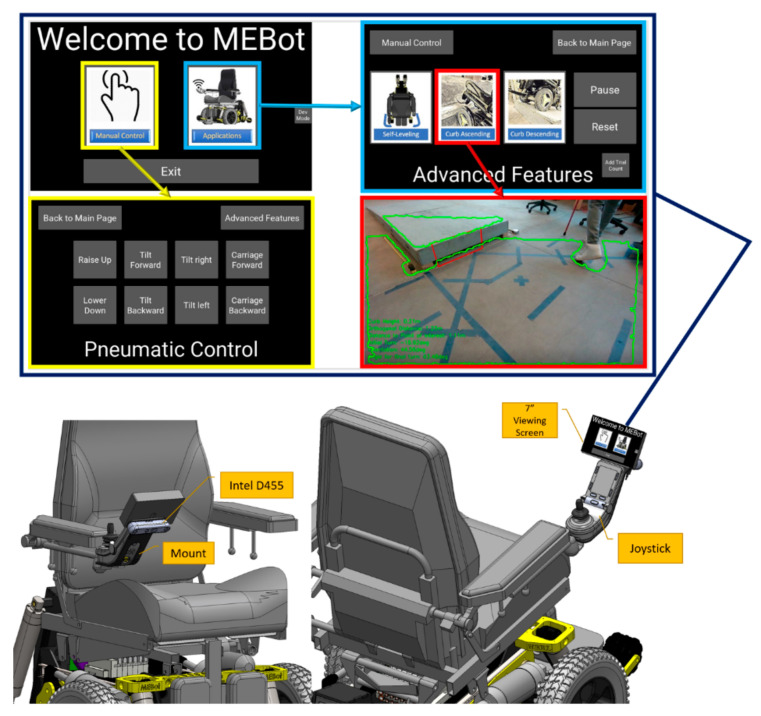
Intel RealSense mounts onto the wheelchair joystick and graphical user interface.

The CRN system uses a pre-planning algorithm to obtain curb characteristics, localization, and orientation towards the curb to assist users and to prepare for climbing while approaching a curb [30]. The CRN system is composed of an Intel D455 camera connected to a PC with a Linux-based operating system (Intel Xeon E-2186M Six Core Xeon 2.90 GHz, NVIDIA Quadro P2000 with 4 GB GDDR 5 and 64 Gb Ram) for curb characterization and a graphical user interface (GUI) for visual feedback. The D455 offers a 90° × 65° field of view for the depth sensor and RGB output of up to a 10 m range. The D455 camera is mounted on a rigid aluminum bracket behind the wheelchair joystick (Figure 2) without compromising the footprint of the wheelchair or the ability to perform common activities of daily living. The GUI is mounted in line with the joystick for adequate visibility and accessibility. The GUI displays power seating functions, such as EPWs, and advanced applications such as self-leveling for tip prevention and curb ascending and descending for accessibility (Figure 2). When either the curb ascending or descending application is selected, the GUI displays the curb ahead and curb parameters obtained from the D455 camera. 

**Figure 3:**
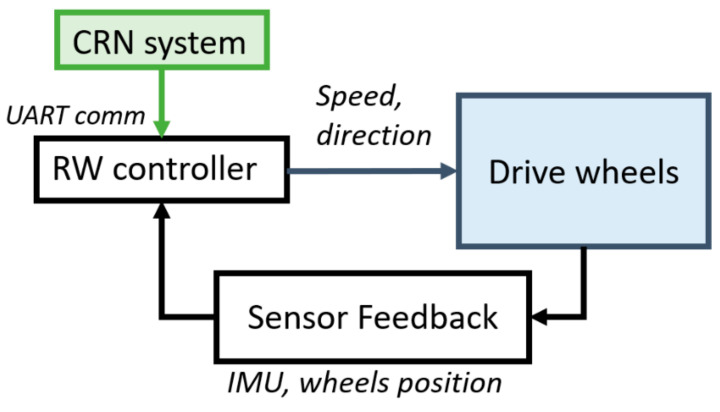
Electronics architecture of CRN and MEBot controller.

The CRN system is integrated into the MEBot controller with serial communication to feed the curb characteristics and angle approach and generates a path to approach the curb. The MEBot controller includes a Teensy 3.6 microcontroller (primary computer) with a 180 MHz ARM Cortex-M4 processor to compute the advanced mobility application algorithms. The algorithm output is fed to a Raspberry PI 3.0 B+ embedded system Cortex-A53@1.4GHz (secondary) (Raspberry PI Foundation, Cambs, UK) attached to an R-net module (Curtis–Wright, PA, USA) to control the speed of the wheel drive motors. Multiple sensors, such as a 9-DOF inertial measurement unit (IMU) and incremental encoders (US-Digital, WA, USA) attached to the drive wheels, measure the wheelchair pitch/roll/heading and position/speed of the drive wheels, respectively. These values are fed back to the MEBot controller to maintain the desired path planning (Figure 3). 

### 2.3. Preliminaries

The purpose of this section is to define 3D points, geometric lines, and reference frame transformations used in the CRN system. A 3D point *p* is defined in a Cartesian reference frame by orthogonal bases ${\hat{e}}_{x},{\hat{e}}_{y},$ and ${\hat{e}}_{z}$:

(1) $$p=x\;{\hat{e}}_{x}+y\;{\hat{e}}_{y}+z\;{\hat{e}}_{z}=\left[x,y,z\right]$$

A 3D point cloud is an arbitrarily ordered array of points denoted as $P=\left\{p_{0},\;p_{i},\;‥,p_{n-1}\right\}$ with an index $i\;\in \left[0,n-1\right]$. This paper follows the Open Geospatial Consortium (OGC) standard [31] for defining a linear ring and polygon. A linear ring is a consecutive list of points that create nonintersecting line segments that join to form a closed path. The key components of a valid polygon are a single exterior linear ring representing the shell of the polygon and a set of linear rings (possibly empty) representing holes inside the polygon. 

A geometric line in a Cartesian frame is defined as the set of all points whose coordinates $\left(x,y\right)$ satisfy the linear equation where a, x, and c are line-specific parameters.

(2) $$l=\{\left(x,y\right)\;|\;ax+by=c\}$$

**Figure 4:**
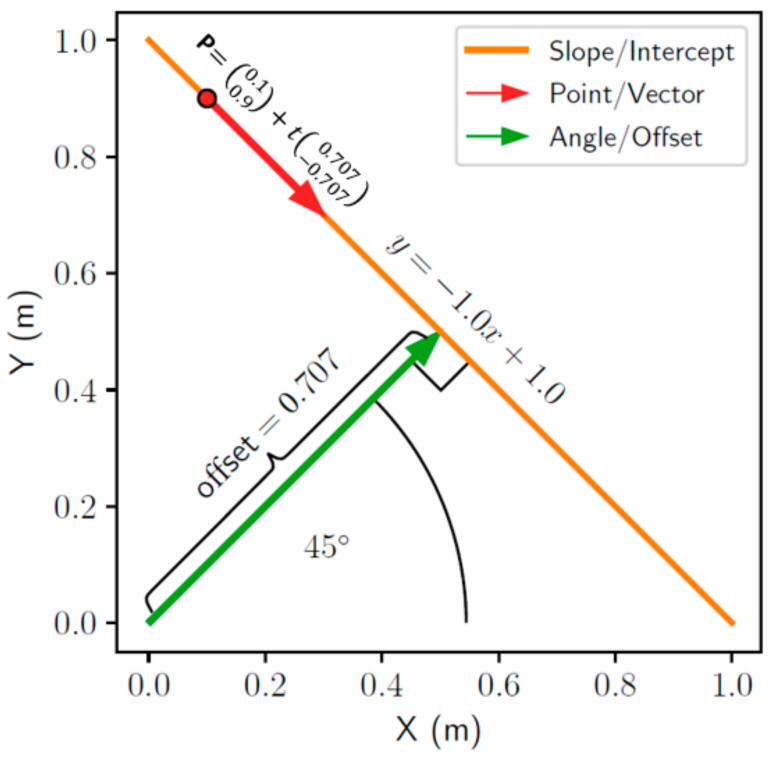
Visualization of three models of the same geometric line.

A geometric line may be represented in alternative forms but satisfy Equation (2) as shown in Figure 4. For example, the ubiquitous slope–intercept form Equation (3) is used often and shown as the orange line parameterized by m and b, respectively.

(3) $$\begin{array}{c} y=mx+b \end{array}$$

Alternatively, the vector Equation (4) may be used to describe the same line. This model has two parameters, any point $p_{0}$ on the line and a “direction” vector v of the line. This is denoted as the red vector in Figure 4.

(4) $$p=p_{0}+vt$$

Finally, the Hesse normal form, which describes the line segment drawn from the origin perpendicular to the line of interest, is expressed as Equation (5) where θ and d are the angle and origin offset, respectively. This representation is shown as the green line in Figure 4.

(5) $$\begin{array}{c} x\cos\left(\theta \right)+y\sin\left(\theta \right)-d=0 \end{array}$$

**Figure 5:**
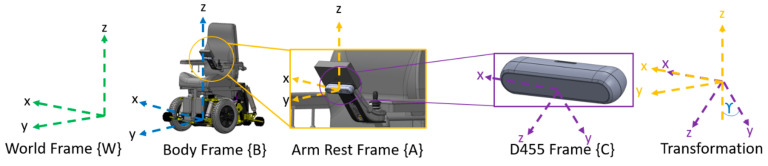
Rigid body transformations.

The CRN system obtains the curb parameters by first defining rigid body transformations as shown in Figure 5. The reference frames used are: World Frame {W}, MEBot Body Frame {B}, armrest frame {A}, and the Intel D455 camera {C}; {B} and {A} are offset from each other and experience no rotation with respect to {W}. The transformation shown in Figure 5 is between {A} and {C} where a rotation of γ will align the frames as given by homogenous transformation (ϒ). All maneuvers and calculations are calculated with respect to the body frame {B}; {B} is located between the two wheels of the MEBot as this is the center point of rotation. In this automated pre-planning process, the MEBot will function primarily in front-wheel-drive; therefore, {B} is in a fixed position.

### 2.4. Curb Negotiation and Classifying the Ground and Sidewalk Planes

Typically, a clear sidewalk would extend beyond the peripheral vision of the user and most depth cameras on the market. However, environmental factors such as snow and parked vehicles may create a small available area to negotiate curbs, not always visible to the human eye. In this research, a curb is classified as a step that extends beyond the field of (camera) vision or a step wide enough for the wheelchair to safely ascend the curb.

When approaching a curb, three dominant surface planes can distinctly define a curb: the ground plane where the MEBot is situated (current location), the sidewalk plane (desired location), and the curb face plane. The MEBot is equipped with an Intel RealSense Depth Camera using Polylidar3D software for plane extraction [28]. Polylidar3D is an open-source application used to extract nonconvex polygons to signify dominant planar surfaces from 3D point clouds. In this research, these polygons were extracted into a line model to define a curb face.

In a perfect scenario, the curb face plane would be the ideal surface for Polylidar3D to extract to compute a curb mounting procedure. However, this surface is often relatively small and may not be reliably extracted by Polylidar3D in certain conditions, e.g., poor lighting, snow/debris obstructions [32], and nonideal camera angles. An attempt to extract the curb height and orientation of such planes may be inaccurate and less reliable in real-life scenarios. Therefore, the curb face plane is inferred from the edge of the sidewalk plane as described below.

**Figure 6:**
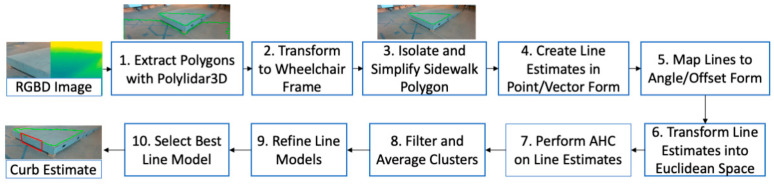
Procedure for extracting a curb estimate from an RGBD image.

Figure 6 outlines our procedure for estimating the curb face plane. First, the Intel RealSense D455 camera provides an RGBD image of the curb. This image is then processed by Polylidar3D [33] to extract all flat surfaces as polygons, which are shown as the green lines in Step 1. In this example, two polygons were returned, the ground surface and sidewalk surface. Each polygon is represented as an ordered list of 3D points that are guaranteed coplanar to a configurable error threshold using Polylidar3D software [33]. All polygons extracted are in the camera reference frame {C} but must be transformed into the MEBot body reference frame {B}. Next, the sidewalk surface must be specifically identified from the list of polygons. This is done by choosing the polygon that is elevated above all other polygons, based on the *z*-axis polygon offset. Next, the sidewalk polygon is simplified by removing redundant vertices in the polygon that are less than 5 cm from each other [34]. The result is a simplified polygon of the sidewalk surface, as shown in Step 3.

**Figure 7:**
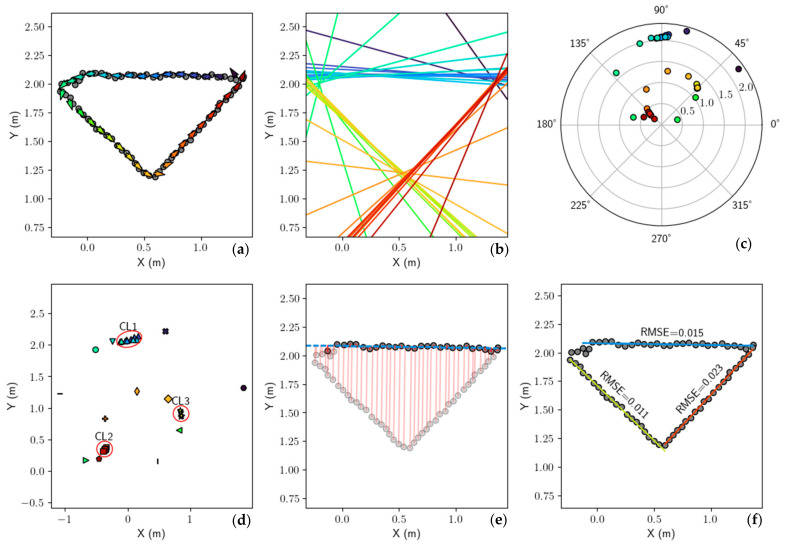
Creating refined line estimates of a polygon. (**a**) Each line segment of the polygonal chain is converted to point/vector form. Color denotes the position in the polygonal chain and is kept constant throughout all plots. (**b**) Vectors shown as full line estimates for better visualization. (**c**) Vectors are converted to Hess normal form whose parameters (angle/offset) are shown on a polar plot. (**d**) The points are transformed to Euclidean space and clustered using AHC. Three clusters are circled in red, which pass constraints. (**e**) The mean of each cluster (only blue shown) is evaluated against all data and inliers are used to refit a final least-squared fit line. (**f**) Final line set returned.

Steps 4–8 describe the method of identifying the curb surface through a series of line estimates, clustering, and model refinement. Figure 7 provides detailed visualizations for each of the steps, starting with the simplified polygon previously shown as Step 3 in Figure 6. Step 4 begins by generating line estimates in point/vector form using line segments of the polygon shown in Figure 7a. Each line segment of the polygon is converted to a unit vector $v_{i}$ while the midpoint of the segment is the identifying point $p_{0}$. These vector estimates are then smoothed by neighboring vectors by applying a uniform filter with a configurable window size, which is set to four in this work. For visualization purposes, each vector (corresponding to a line segment) is colored according to its index position in the polygon chain. This allows line estimates that are near each other to have a similar color and allows the reader to “track” a line through Figure 7a–f. Figure 7b shows the same vectors in their full line form and utilizes the same color-coding. Step 5 then maps from point/vector form to Hess normal form. Figure 7c displays these lines which are parameterized by angle/offset and plot in polar coordinates. Now in parameter space, it becomes clear that similar line estimates (often near in hue) appear close together on the polar plot.

The mapping from line form to angle/offset parameter space is similarly performed during a Hough transform [35]. This technique is often used to estimate lines in a raster image by detecting edge points in an image and transforming them to continuous curves in the Hough parameter space. However, our method directly transforms the line estimates to points in parameter space as seen in Figure 7c. Clustering techniques may now be used with these points to identify a set of lines that best fit the complete dataset. In this specific example, there are three lines that best fit the data: one each for the red, blue, and yellow line sets. It is important to note that the number of lines k is unknown beforehand as the camera perspective and sidewalk may change as the MEBot approaches curbs. This makes line clustering techniques such as k-means [35] and k-median [36,37] more difficult to use and computationally intensive as multiple values of k must be evaluated. For these reasons, we decided to investigate other clustering techniques.

The Hough parameter space does not prove a valid metric distance calculation between points that is required for clustering. We resolved this issue in Step 6 by converting the polar coordinates (parameters) to their Cartesian representation (x=rcos(θ), y=rsinθ) as seen in Figure 7d. The line estimates were mapped as points in Euclidean space, which can use the L2 norm for distance calculations. In Step 7 we performed agglomerative hierarchical clustering (AHC) on the points using a single linkage distance of δ=10 cm [38,39]. AHC does not require knowledge of k beforehand and gave excellent results in our tests. Figure 7d represents the cluster groups found in this step by marker shape while the color of each point is held constant from previous plots. Step 8 then performs cluster filtering where any cluster whose cardinality is less than three is ignored and considered invalid. In the example presented, only the blue triangles, red squares, and yellow stars meet these constraints and are denoted CL1, CL2, and CL3, respectively. The mean of each cluster is then computed and transformed back to polar coordinates to their Hess normal line representations. For example, the dashed blue line in Figure 7e is the blue triangles cluster (CL1) average. Step 9 then refines each mean line model by first calculating the orthogonal distance of all points to the line shown as red lines in Figure 7e. Similar to RANSAC [40], a point is considered an inlier if the orthogonal distance is less than a configurable ε, set to 5 cm in this work. A new line is then refit using linear regression using only the inliers to provide a more robust estimate. Outlier points and their associated orthogonal distances are shown as slightly transparent in Figure 7e. Finally, any line model that has an inlier ratio of less than 15% is ignored, ensuring that only the large dominant edges of the curb are recovered. Figure 7f shows the final three candidate line model set, denoted L, and their RMSE values.

In Step 10, a single line must be chosen from the candidate set to select the curb surface for mounting. This is done by identifying the line that is nearest to the MEBot body frame {B} origin and almost perpendicular with respect to the MEBot heading. We found that optimizing for these objectives selected a mounting surface that is most accessible to the MEBot. The distance to the line is calculated from the mean point of all data points used to fit the line (the inliers), which we denote as $\mu_{i}$. We formulate this as an optimization problem with both objectives equally weighted, as in Equation (6), where $v_{i}$ and $h_{w}$ denote the line direction vector and MEBot heading, respectively.

(6) $$\begin{array}{c} l_{i*}=\underset{l_{i}\;\varepsilon \;L}{\arg min}0.5\;\cdot \;\left(v_{i}\;\cdot \;h_{w}\right)+0.5\;\cdot \;||\mu_{i}|| \end{array}$$

In this example, the yellow line was chosen because it was significantly closer in distance and alignment to the MEBot body frame {B}. The curb surface normal $n_{c}$ was then inferred from this line by taking the cross product of the curb edges line vector and the ground plane normal. The height of the curb was calculated as the elevation difference between the ground floor and sidewalk polygon. The bottom left image of Figure 6 shows the final curb surface estimate as a red rectangle.

### 2.5. Path-Planning Algorithm

The curb negotiation process from prior work assumed a pre-defined curb height and a perpendicular approach angle towards the curb (Figure 1). These steps were performed manually by the user on an engineered curb. To prevent human error during wheelchair alignment, the CRN system was integrated into the MEBot controller to obtain the curb characteristics and generate a path to align the MEBot perpendicular to the curb. 

**Figure 8:**
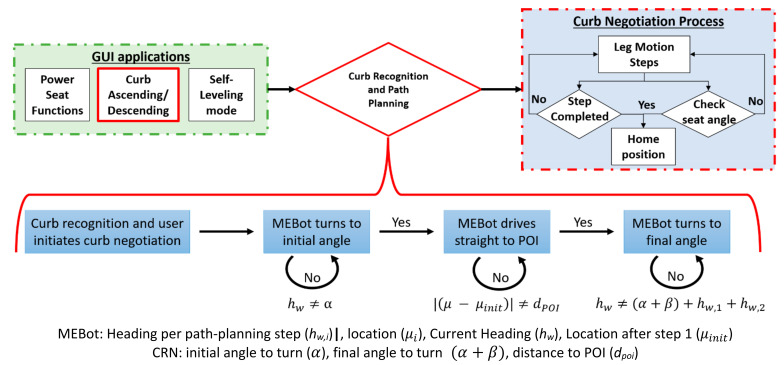
Current curb negotiation process of the MEBot (top). Improved process using CRN system (bottom).

The CRN system outputs the curb parameters (curb height, distance, orientation, and angle to the curb) into the MEBot controller via serial communication at a 10Hz sampling rate. Parameter collection begins one second after the user initiates the curb negotiation application (Figure 8). This action is performed to reduce computing power and prevent redundant information from being fed to the MEBot controller. The parameters are filtered through a moving average filter to reduce noise and outliers. Afterward, the parameters are used to calculate the path to perform automated navigation towards the desired point of interest (POI). The POI was defined as the center point of the desired curb width and 0.7 m offset from the curb. This was the required distance to start the curb negotiation process as established in previous work [41]. 

The control commands needed to maneuver the MEBot towards the POI were determined by first calculating the POI location in Equation (6), where $\mu_{c}$ is the center of the curb edge and δ is a configurable offset from the curb, set to 0.7 m in this work.

(7) $$\begin{array}{c} \mathrm{POI}=\mu_{c}+\delta \;\cdot \;n_{c} \end{array}$$

**Figure 9:**
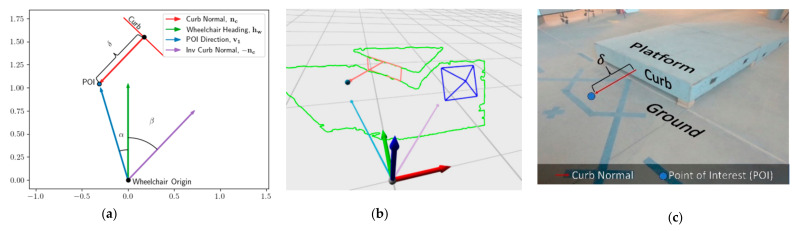
(**a**) Two-dimensional planar, (**b**) three-dimensional, and (**c**) image representations of the path-planning process, where the POI is the point at where the MEBot will arrive and is offset from the curb by δ. The dark blue lines in (**b**) represent the RealSense position and field of view.

A vector $v_{1}$ is calculated as the difference between the POI and MEBot origin. The path plan is then computed geometrically through a composition of three movements: an initial turn α that aligns the MEBot heading to the POI, a distance traversal $||v_{1}||=d_{POI}$ such that the MEBot origin is coincident with the POI, and finally a turn α+β such that the MEBot heading $h_{w}$ is parallel with the curb surface normal $n_{c}$. These geometric quantities are displayed in Figure 9a while their 3D representation is shown in Figure 9b,c. The path-planning algorithm outputs the desired speed and direction of the motors of the drive wheels to reach the desired heading and position of the wheelchair.

### 2.6. Experimental Protocol

**Figure 10:**
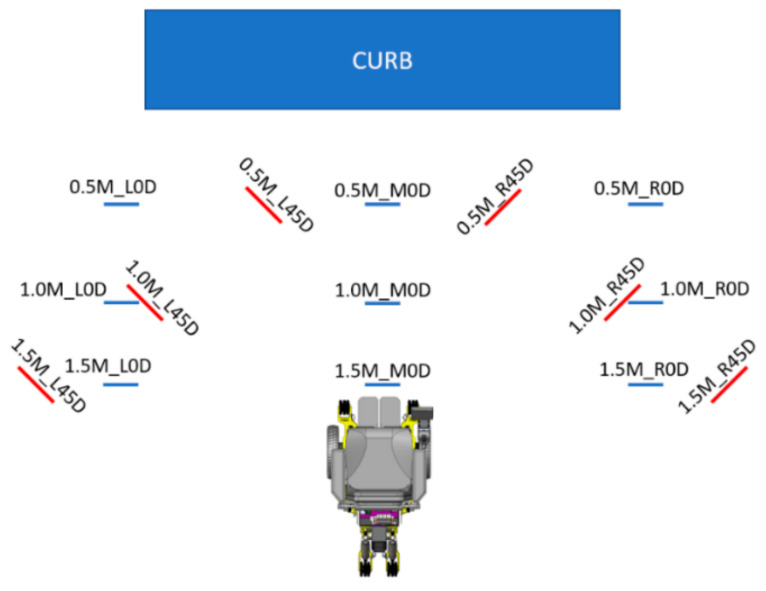
MEBot curb negotiation protocol diagram at 0.5–1.5 m away from the curb.

We evaluated the accuracy of the CRN system to measure the curb characteristics and different angle approaches towards an engineered curb in controlled lighting conditions. The efficacy of the CRN was also evaluated when navigating towards the curb based on the planned path. The platform modeled as a curb measured 1.22 m by 1.22 m (4 ft × 4 ft) with a height of 0.20 m (8 inches). A 50th percentile Hybrid II anthropometric dummy of 100 kg was used to simulate a person situated in the MEBot. Three trials were performed at each of the 15 starting positions as shown in Figure 10, resulting in a total of 45 trials. The testing protocol was conducted from a 0.5 to 1.5 m offset from the curb, measured from the center of the driving wheels to the curb face. If a user needed to climb a curb and a car was blocking the curb cut (given an average car width of 1.7 m), then a 1.5 m distance would be sufficient to signify intent to climb a curb. Each starting position is marked in Figure 10 by the distance (meters), location (left, middle, and right), and angle to the curb (“0.5M_R45D = 0.5 Meters_ Right side angled at 45°”) (Figure 10). During each trial, the CRN system recognized the curb, generated a path based on curb characteristics, and proceeded to orient the MEBot perpendicular to the curb to then initiate the curb negotiation process. The MEBot’s front driving wheels were aligned to each of the preset positions at the start of each trial. The MEBot average speed was set to 1.2 m/s, which matches the average person’s walk speed to cross the street [42].

### 2.7. Data Analysis

The accuracy of the CRN system to recognize curb characteristics was evaluated by calculating the mean and standard deviation of three trials for the outcome variables: curb height and orientation toward the curb. Results of each outcome measure were compared to a pre-defined curb height of 0.21 m (8.0 inches) with preset angle approaches toward the curb.

The efficacy of the CRN to complete a suggested path plan towards the curb was evaluated during its four steps as shown in Figure 10. The efficacy was evaluated by comparing the average and standard deviation error rate to reach the initial turn (Step 1), distance to POI (Step 2), final turn (Step 3), and final distance to curb (Step 4). The least amount of misalignment in each step would ensure a safe curb negotiation process. All statistical analysis was performed with Microsoft Excel. 

## 3. Results

**Figure 11:**
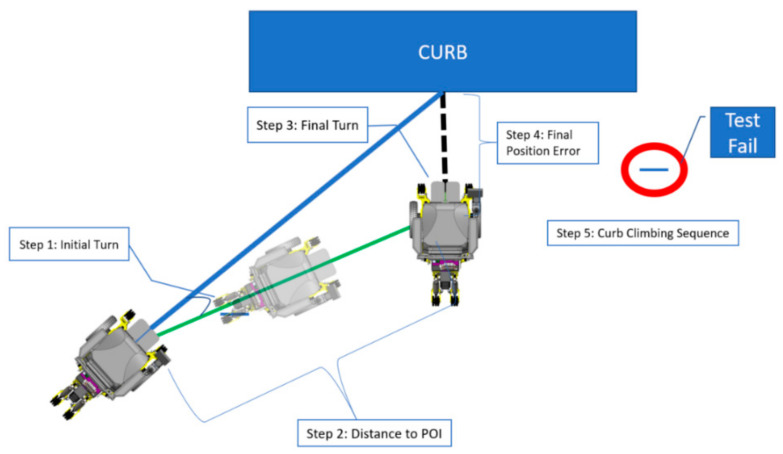
Automated curb negotiation process from a tested angle approach. Starting position “0.5M_R0D”, highlighted in red, indicated a test failure.

**Figure 12:**
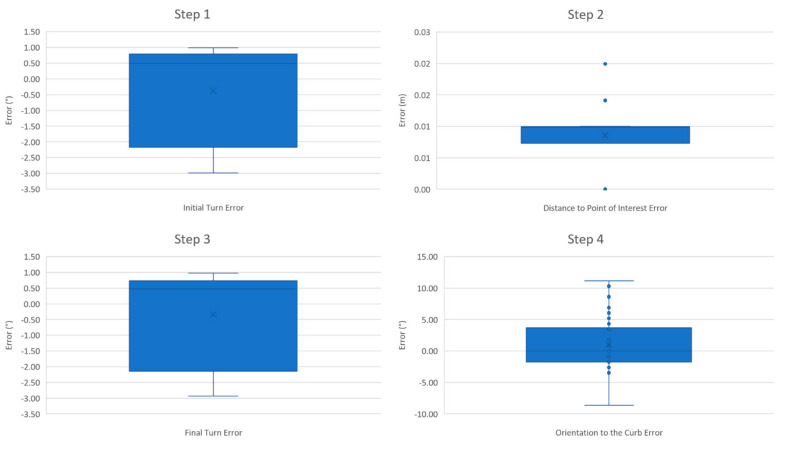
Box and whiskers error plots of the stepwise process.

Over the 15 starting positions spanning the platform, the CRN system correctly detected the curb at 14 starting positions (42/45 trials) and failed to detect the platform 0.5 m perpendicular to the curb and 1.0 m away to the right of its center (“0.5M_R0D”, highlighted in red on Figure 11). In this final case for each of the three trials, the curb negotiation procedure could not be activated. All three trials for this position failed and there were no other failure trials. Curb height detected by the CRN system showed a high accuracy at all other 14 starting positions. Table 1 highlights the average time to complete each step and the average maneuvering and turning error at each step. The overall time and maneuvering error of the trials combined in each step are displayed. The overall average time to perform the path-planning navigation towards the POI was 13.46 ± 2.33 s. Furthermore, the average time to ascend the curb was 54.99 ± 13.86 s (Table 1). To illustrate the turning errors at each step from Figure 11 and corresponding values in Table 1, a box and whiskers plot is shown in Figure 12. The highest variation of error occurred at Steps 3 and 4; these are represented as outliers in Figure 12, when the MEBot made its final turn to align perpendicular to the curb. The standard deviation for the time taken to maneuver the MEBot is stated in Table 1 for positions “0.5M_L45D”, “1.0M_L0D”, ‘0.5M_L0D”, ”1.0M_R45D”, and “0.5M_R45D”.

## 4. Discussion

### 4.1. Perception-Based Reliability

**Figure 13:**
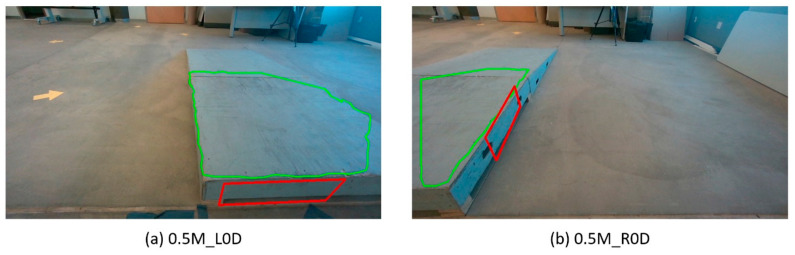
(**a**) Left side and (**b**) right side of the curb for curb detection comparison. The CRN system failed to detect the front of the curb in (**b**). Note that the RealSense camera is mounted on the right side of the MEBot, thus limiting its field of view.

Our CRN system demonstrated the ability to detect the curb dimensions at different approach angles. The curb height was calculated based on a moving average and discarded anomalies; therefore, it was accurate every time in controlled lighting conditions. Only 1 out of 15 different starting positions was not successful due to the location of the camera mounted on the joystick on the right side of the MEBot, which limited its field of view. The anomalies stated with a high standard deviation in timings at “0.5M_L45D”, “1.0M_L0D”, ‘0.5M_L0D”,”1.0M_R45D”, and “0.5M_R45D” were due to a battery discharging and causing the MEBot to slow down in terms of performance; however, the accuracy of each maneuver was not affected. Figure 13 shows a comparison between the left- and right-hand sides of the curb to illustrate how the front part of the curb is not visible for the case “0.5M_R0D”. Although the other side of the curb posed another mounting option for the MEBot, it was deliberately excluded as this may not normally be a viable option in outdoor scenarios. 

One camera was used for the study to verify the maximum potential; additional cameras could increase the CRN recognition capabilities and in turn increase the computation power requirements. Therefore, further testing is required to characterize the impact of this limitation for EPW users. Polylidar3D used in the CRN system was designed to detect flat surfaces and reliably identified the planar surfaces in the experiments. 

It is known from other research that Intel RealSense depth sensor noise grows quadratically with distance [43], meaning that the farther the sensor is positioned from the curb, the lower the accuracy of the curb detection will be. On the other hand, our findings showed high accuracy up to 1.5 m away from the curb where the user could have the option to activate the CRN system ahead of time. It is noted that the current CRN system assumes a straight curb edge and is unable to detect curbs with rounded corners.

### 4.2. Pre-Planning Process

The CRN system verified the ability of a pre-planning process to automatically approach the curb prior to commencing the curb negotiation application. This was shown by a successful demonstration, as three trials were completed at each starting position and only one position did not detect the curb. As a safety and efficacy protocol, this is beneficial to prevent curb misalignment.

Current EPWs are not able to negotiate curbs and while several RWs offer step-climbing capabilities, these devices require proper alignment to the curb, usually performed by the user. This factor adds human error, in addition to machine error for curb misalignment. The CRN system balances the RW’s limitation for proper curb alignment by reducing human error to safely approach a curb. While the feasibility of the CRN system was evaluated, another factor to consider is an adjustable level of autonomy suggested by the user. It is necessary to understand the appropriate distance at which end-users could decide to negotiate curbs in real-life scenarios. 

The capabilities of the CRN system can be expanded to common EPWs to detect and avoid obstacles. This allows people with lower cognition or visibility to safely maneuver an EPW, thus increasing their autonomy. The curb negotiation process can be tailored uniquely to suit those with cognitive, visual, or severe mobility impairments who might also require caregiver support. 

There are a few outliers in Table 1 and displayed in Figure 12 for negotiating on the right side of the curb, at which the maneuvering errors are high. This could be because the camera was mounted on the right side of the joystick, and future work will include investigating these errors. On the other hand, although there were curb orientation alignment anomalies, the MEBot was still able to safely negotiate the curb. There were certain trials of the curb climbing process when the time required was significantly less, which was due to the air tank pressure not being the same for each trial. In the future, either the tanks need to be maintained at higher pressures or an alternative power system should be developed. From a user’s perspective, these usability evaluations provided mixed responses whereby some preferred a faster process whilst others preferred the safety and reliability of the MEBot. These challenges were identified in other usability evaluations [29,30]. Further work will include modifying the curb climbing sequence to reduce the time taken to climb a curb.

### 4.3. Future Work

There is a need to test the system under different environmental conditions for this perception-based method. Polylidar3D does detect obstacles as interior holes and could be used to identify sections of the curb that are obstacle-free. Moreover, a surface area threshold can be set to identify sufficient space so that the user can safely negotiate the curb. Bright lighting conditions will saturate the imaging sensors on the Intel D455, but these sensors can set an auto-exposure control to limit the amount of light retained. Further testing is required to determine if one sensor is sufficient or if additional sensors are required to accurately adjust between indoor and outdoor lighting conditions. Another option is to classify the curbs and the type of terrain (mud or grass) by analyzing the terrain color or using deep learning models. The platform used to model a curb had a straight small curvature for its edge, and there can be scenarios where that might not be the case. Therefore, more testing on more curbs of different heights and with obscure nonuniform edges can verify the reliability of the recognition algorithms. The battery discharging issues slowing the performance of the MEBot will be investigated. This will ensure that it can consistently perform throughout the day using all of the advanced applications. 

Finally, the path-planning algorithm was a simple three-step process that may not be the shortest path to navigate to the POI. Therefore, other shortest path algorithms could be implemented and tested to verify reliability and algorithm execution time. The current computational device used was a laptop due to its computing power. Future work will include translating this work into a smaller embedded system design so that it can be securely mounted within the base of the MEBot.

## 5. Conclusions

This study introduces a CRN system applicable to RWs, designed to assist an automated curb negotiation process up to 1.5 m away from the curb. The reliability and efficacy evaluation using a 50th percentile Hybrid II anthropometric dummy of 100 kg provides evidence of the CRN system’s capabilities to recognize curb dimensions and demonstrated an automated path-planning navigation solution to approach and negotiate curbs. The study was performed in a laboratory setting; further evaluation is required to test its reliability in real-world environments. Future studies will be complemented with end-user feedback to evaluate the feasibility of the CRN system to navigate challenging environments and improve the mobility and autonomy of EPW users.

## 6. Patents

Authors: Rory Cooper and Jorge Candiotti are patent holders of the Mobility Enhancement Robotic Wheelchairs (US20210128378A1).

## Figures and Tables

**Table 1:** Stepwise average maneuver error and time taken for 3 trials at each of the 15 positions.

Starting Position	Step 1		Step 2		Step 3		Step 4		Step 5
	Initial Turn		Distance to POI		Final Turn		Final Position		Curb Climbing
	Error (°)	Time (s)	Error (m)	Time (s)	Error (°)	Time (s)	Error (m)	Orientation Error (°)	Time (s)
1.5M_L45D	0.57 ± 0.17	1.81 ± 0.03	0.01 ± 0	4.63 ± 2.67	0.61 ± 0.28	4.82 ± 0.64	0.15 ± 0.04	−4.03 ± 3.97	64.20 ± 0.48
1.0M_L45D	0.74 ± 0.22	2.35 ± 0.03	0.01 ± 0	4.22 ± 2.12	0.77 ± 0.06	7.67 ± 2.1	0.2 ± 0.01	1.44 ± 4.28	63.84 ± 1.22
0.5M_L45D	0.66 ± 0.21	6.96 ± 2.51	0.01 ± 0	3.63 ± 2.21	0.6 ± 0.24	8.22 ± 1.34	0.26 ± 0.03	−1.45 ± 0.50	59.99 ± 1.24
1.5M_L0D	0.79 ± 0.08	3.46 ± 0.25	0.01 ± 0.01	3.59 ± 0.06	0.54 ± 0.15	6.7 ± 2.21	0.03 ± 0.01	−0.87 ± 0.87	64.05 ± 3.6
1.0M_L0D	0.83 ± 0.19	5.22 ± 0.35	0.01 ± 0	3.20 ± 0.11	0.66 ± 0.05	8.77 ± 3.46	0.04 ± 0	−1.45 ± 1.33	50.32 ± 25.65
0.5M_L0D	0.54 ± 0.29	7.21 ± 3.12	0.01 ± 0.01	2.68 ± 0.03	0.68 ± 0.31	8.62 ± 1.8	0.18 ± 0.03	−2.02 ± 1.00	54.85 ± 13.22
1.5M_M0D	−0.15 ± 0.54	2.09 ± 1.2	0.01 ± 0.01	2.89 ± 0.03	0.60 ± 0.27	1.4 ± 0.13	0.02 ± 0.02	0.58 ± 1.00	49.56 ± 19.84
1.0M_M0D	0.86 ± 0.09	0.97 ± 0.14	0.01 ± 0.01	3.38 ± 2.06	0.76 ± 0.21	2.04 ± 1.34	0.04 ± 0.03	−2.60 ± 0.87	38.32 ± 22.17
0.5M_M0D	0.76 ± 0.07	7.45 ± 0.24	0 ± 0.01	1.33 ± 0.13	0.77 ± 0.22	9.06 ± 2.15	0.15 ± 0.01	−0.87 ± 0.87	42.19 ± 16.66
1.5M_R0D	−2.62 ± 0.22	3.83 ± 0.72	0.01 ± 0.01	3.83 ± 0.08	−2.63 ± 0.17	3.54 ± 0.11	0.07 ± 0.05	0.29 ± 0.50	53.87 ± 12.22
1.0M_R0D	−0.66 ± 0.18	3.05 ± 0.19	0 ± 0.01	3.46 ± 0.12	−0.61 ± 0.22	4.25 ± 2.08	−0.14 ± 0.06	8.04 ± 2.72	66.21 ± 1.99
0.5M_R0D	TEST FAIL								
1.5M_R45D	−2.52 ± 0.44	2.35 ± 0.44	0.01 ± 0.01	3.62 ± 0.17	−2.59 ± 0.41	5.38 ± 1.64	0.04 ± 0.06	5.76 ± 2.62	60.15 ± 1.72
1.0M_R45D	−2.79 ± 0.25	5.04 ± 1.71	0.01 ± 0.01	2.54 ± 0.14	−2.73 ± 0.06	9.32 ± 3.96	0.04 ± 0.03	4.04 ± 2.78	59.82 ± 7
0.5M_R45D	−2.34 ± 0.26	4.24 ± 1.71	0.01 ± 0	2.34 ± 0.49	−2.19 ± 0.38	7.20 ± 6.25	0.18 ± 0.03	7.16 ± 5.45	42.44 ± 8.58
Overall avg ± stdev	−0.38 ± 1.48	4.00 ± 2.33	0.01 ± 0.01	3.24 ± 1.30	−0.34 ± 1.46	6.21 ± 3.37	0.09 ± 0.10	1.00 ± 4.26	54.99 ± 13.86

## Data Availability

The data presented in this study are available on request from the corresponding author. The data are not publicly available due to them being restored by the US Department of Veterans Affairs and subject to approval of the relevant authority.
